# Social factors and behavioural reactions to radon test outcomes underlie differences in radiation exposure dose, independent of household radon level

**DOI:** 10.1038/s41598-022-19499-5

**Published:** 2022-09-14

**Authors:** Jesse L. Irvine, Justin A. Simms, Natasha L. Cholowsky, Dustin D. Pearson, Cheryl E. Peters, Linda E. Carlson, Aaron A. Goodarzi

**Affiliations:** 1 Departments of Biochemistry & Molecular Biology and Oncology, Cumming School of Medicine, Robson DNA Science Centre, Charbonneau Cancer Institute, University of Calgary, Alberta, Canada; 2 Faculty of Medicine, University of Saskatchewan, Saskatoon, Saskatchewan Canada; 3 British Columbia Centre for Disease Control, British Columbia Cancer Agency, School of Population and Public Health, University of British Columbia, British Columbia, Canada; 4 Division of Psychosocial Oncology, Department of Oncology, Cumming School of Medicine, Charbonneau Cancer Institute, University of Calgary, Alberta, Canada

**Keywords:** Environmental impact, Cancer prevention, Lung cancer

## Abstract

Radioactive radon gas inhalation causes lung cancer, and public health strategies have responded by promoting testing and exposure reduction by individuals. However, a better understanding of how radon exposure disparities are driven by psychological and social variables is required. Here, we explored how behavioural factors modified residential radon-related radiation doses incurred by 2390 people who performed a radon test. The average time from first awareness to receiving a radon test outcome was 6.8–25.5 months, depending on behaviour and attitudes. 20.5% displayed radon test urgency that reduced irradiation between awareness and outcome to 1.8 mSv from a typical 3.5 mSv, while 14.8% (more likely to be men) displayed delaying behaviours that increased exposure to 8.0 mSv. Of those with low radon, 45.9% indicated no future testing intention, underscoring the importance of original tests to reliably establish risk. Among people finding high radon, 38% mitigated quickly, 29% reported economic impediments, and 33% displayed delaying behaviours. Economic barriers and delaying behaviours resulted in 8.4 mSv/year or 10.3 mSv/year long term excess exposure, respectively, increasing lifetime risk of lung cancer by ~ 30–40%. Excess radiation doses incurred from behaviour were independent of household radon level, highlighting the strong influence of psychological and socioeconomic factors on radon exposure and lung cancer risks.

## Introduction

Lung cancer is responsible for 1 in 4 cancer-related deaths in North America and Europe, and lung cancer in people who have never smoked is now the 7th leading cause of cancer-linked death globally^1–5^. Radioactive radon gas inhalation in the residential built environment is a principal cause of lung cancer, causing many thousands of new diagnoses per year, including populations who have never smoked tobacco^3,6–15^. The long term inhalation of radon and its airborne decay progeny cause lung cancer by bombarding cells with alpha particle radiation^7,10–16^. Because alpha radiation damages lung DNA to produce genetic mutations that elevate cancer risk, radon is a classified by the *International Agency for Research on Cancer* as a group 1 carcinogen, alongside tobacco, asbestos and air pollution particulate matter^14–16^. Levels of alpha particle radiation from radon within a building is measured in Becquerels (Bq) per cubic meter (m^3^), equal to one radioactive emission per second per cubic metre of air. When generalized across populations, there is an additive 16% increase in relative lifetime risk of lung cancer per 100 Bq/m^3^ of long term radon exposure^17,18^. To calculate absorbed radiation doses for individuals or small groups, however, Bq/m^3^ indoor air radon levels must be integrated with measures of exposure duration (hours per year breathing that air) to derive Sievert (Sv) doses of absorbed energy per mass. The global average alpha radiation exposure from residential radon is currently reported to be 1.2 mSv per year (mSv/year)^19^, but this varies by region and demographics^9,20^. Based on data obtained from the Canadian and American *National Human Activity Pattern Study*, the average North American adult spends 6018 h/year inside a residence^21^. Using the latest conversion formula standardized by the *International Commission for Radiological Protection* (ICRP), this would mean 100 Bq/m^3^ of residential radon equates with 4 mSv/year that increases relative lifetime risk of lung cancer by 16%. Allowing for a 10–30 year period of radon exposure prior to lung cancer diagnosis^4,5,9,10,14^, this estimates 40–120 mSv as the minimum range of absorbed alpha particle radiation from radon that is needed to increase lifetime lung cancer relative risk by the minimum statistically significant amount over the long term; this range is also in agreement with observations of radon-induced lung cancer using rodent models^22^.

It is important to note that prevalent, unsafe radon levels in indoor air is a modern, human-made problem largely rooted in the design of our built environment. Although radon is emanated by most of the Earth’s subsurface, through most of human history it is reasonable to hypothesize that it diluted naturally in the atmosphere, or was effectively vented from (predominantly non air-tight) buildings to low (< 100 Bq/m^3^) levels with no evident health impacts^14^. Regrettably, construction practices over the past century have produced urban and rural environments with buildings that capture, contain, and concentrate radon to unnatural and unsafe levels^12,13,20,23^. Residential radon gas levels continue to change over time, often as a function of evolving regional building trends. For example, new Canadian houses currently show 467% higher radon levels vs modern Swedish equivalents, although radon levels in mid-to-late twentieth century Canadian properties were either equivalent to or less than those in Sweden^23^. This scenario is thought to have arisen not from any identifiable, specific radon-related intervention, but rather as a collateral consequence of ever-changing and often diverging region-specific building practices.

Aside from building features or local geology, there are also social and economic factors that can influence how different populations are exposed to radon. For example, in many regions of North America, mid-to-late twentieth century properties with comparatively lower radon levels are less accessible to younger people due to high prices, biasing these individuals (particularly first time homeowners with small children) towards living in newer, more affordable communities that have higher radon levels and, thus, producing youth-skewed radon exposure trends driven by socioeconomics^20^. Public health organizations have responded to the global radon exposure crisis by broadly promoting awareness of radon health effects, and encouraging exposure reduction at the level of the individual home-owner^3,6–12^. Given that the onus is on the individual to act to reduce radon exposure, it is worth noting that typical radon and lung cancer risk communication approaches are not operating in an inclusive manner, with issues in messaging identified on the basis of ethnicity, region, education, age, sex, and profession^24–28^.

A majority of public radon-related health strategies focus on getting individuals to test buildings and personally invest in radon mitigation to remove risk. By relying solely on individual action, there is an inherent reliance on individual motivation and capability, influenced by socioeconomic factors and social determinants of health, to act to reduce the health threat of radon exposure^29,30^. This can result in inequitable exposure and hence increased risk for lung cancer in those groups less likely to take personal action to mitigate risk. More specifically, how long it takes someone to first become aware of radon, obtain a test, complete a test, or act (based on the outcome of a test), and whether they do (or are able to afford) any of this at all, are influenced by education, income, information processing capability, emotional reactions, and decision-making to both measure and remove radon as a source of lifetime lung cancer risk. Consider a simple example of a low income family in a basement rental unit struggling with food insecurity and medical expenses—checking and/or mitigating their radon exposure is unlikely to be prioritized above more fundamental needs. The influence of these factors on radon exposure, particularly on doses of alpha radiation experienced at an individual level, are not well understood. To address this, we assessed if emotional reactions, economic barriers, and decision-making following radon test outcomes differed between people who performed long term radon test, and how this impacted long term radiation exposure. Our main objective was to determine whether select social, economic, and behavioural differences were variables that modified radiation exposure, beyond baseline doses understood from household radon levels alone.

## Results

### Study cohort details

A description of the demographic details of this cohort, as well as radon test outcomes and property types were described recently in Ref.^20^. The total study population currently includes Canadians enrolled in the ‘Evict Radon’ national study, a citizen science-based radon awareness and testing program of interdisciplinary research led by investigators at multiple Canadian universities. Participants performed a long-term alpha track radon test within their primary residential property, and consented to provide de-identified radon data and selected property metrics to researchers. In this analysis, 7481 participants active in the study in 2018 were invited to answer retrospective questions (in relation to radon testing) concerning their radon test decision making, emotional reactions to test outcomes, known and intended course of actions following test outcome, personal demographics, and activity patterns as it related to time spent in the radon-tested property per year. Of these, 2390 people provided responses for age, sex, gender, relationship status, parental status, occupants per property, household income, number of years lived in the tested property, whether they had ever performed a radon mitigation on the property, and their activity patterns. Response distributions for the entire group are indicated throughout this study (denoted by ‘ALL’) and have also been previously described in Refs.^20,24^. As we obtained gender identity responses from a majority but not all participants, we defaulted to sex for individuals whose gender data was missing (see “Methods” section). Responses regarding sex had options for “other” or “prefer not to say” that were ultimately chosen by 1% of participants. For ease of reading, we will hereafter refer to this metric as ‘sex/gender’ and will use the terms ‘man’, ‘woman’ and ‘everyone else’. Our cohort comprised 42.9% women (mean age 49), 56.1% men (mean age 53), 1% everyone else (mean age 53), an average of 2.56 occupants per property, 73% parents to least one child, with 44% reporting that minors lived in the property at the time of surveying. As a whole (and prior to the COVID-19 pandemic), the cohort spent 5549 h/year inside their primary residence (data was all collected pre-COVID-19 pandemic), with less time for those in full time work or education (5205 h/year), and more for those retired, telecommuting, or on leave (6200 h/year), or unemployed (6726 h/year).

### Residential radon exposure periods modified by radon awareness and testing behaviour

**Figure 1:**
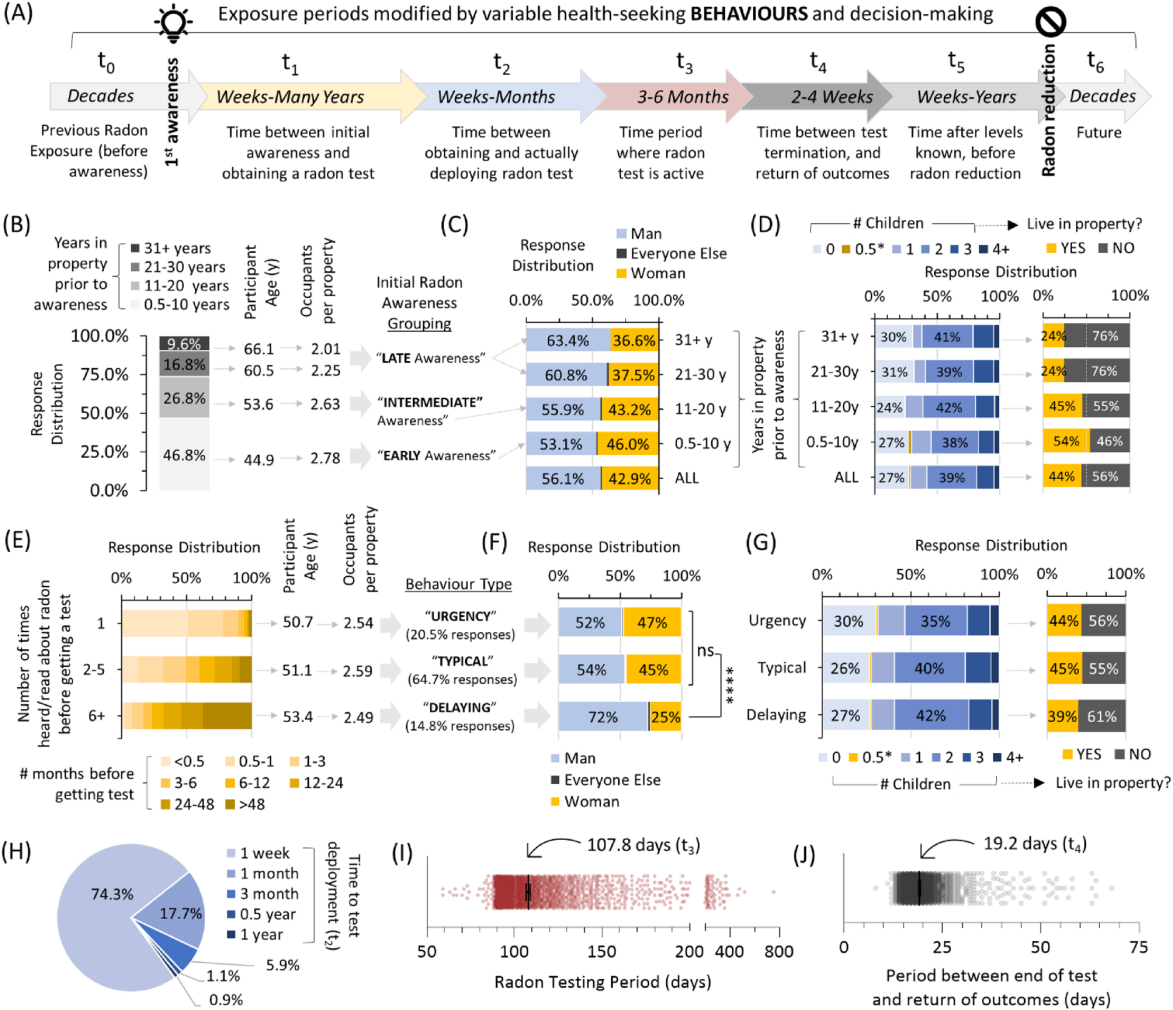
Residential radon exposure periods modified by radon awareness behaviour and test decision-making that can differ by age, sex/gender or parental status. (**A**) A schematic indicating seven variable time periods (t_0_ to t_6_) influencing human exposure to radon gas in residential properties. (**B**) The period from first moving into a primary residence to initial radon awareness (t_0_) separated into early, intermediate and late awareness groups, with geometric mean age and occupants per property. (**C,D**) Sex/gender distributions, total number of children, children living in property at time of radon testing, for the groups outlined in (**B**). 0.5* indicates households with pregnant occupants. (**E**) The period from initial radon awareness to obtaining a radon test (t_1_) separated into urgent, typical and delaying behavioral groups, with geometric mean age and occupants per property. (**F,G**) Sex/gender distributions, total number of children, children living in property at time of radon testing, for the groups outlined in (**E**). 0.5* indicates households with pregnant occupants. Statistical comparisons are 1-way ANOVA. ****p < 0.0001; ns = p > 0.05. (**H**) Distribution of periods from obtaining to deploying a radon test (t_2_). (**I**) Individual radon testing periods (t_3_) with geometric mean. (**J**) Individual radon test return to outcome periods (t_4_) with geometric mean. Figures were prepared using Excel and GraphPad Prism 9.1.1 (225) (www.graphpad.com).

We first defined six radiation exposure time periods of interest, across the spectrum of radon awareness to mitigation behaviors (Fig. 1A). These were: t_0_, the time before initial awareness of radon after moving into primary property; t_1_, the time between first gaining awareness of radon to obtaining a radon test; t_2_, the time taken to deploy the test; t_3_, the radon testing period; t_4_, the time between test conclusion and receiving outcomes; t_5_, time after radon levels are known but before action is taken; t_6_, all periods after action is taken (including a decision to do nothing). For t_0_, we stratified people into three groups based on how long it took them to first hear about radon after moving into their primary residence. 26.4% lived in the property for > 20 years before encountering knowledge of radon gas and its health effects, and, as a group (we denoted as ‘late awareness’), were older with fewer household occupants compared to the 46.8% who gained awareness within the first decade (‘early awareness’) or 26.8% who gained awareness within the second decade (‘intermediate awareness’) (Fig. 1B). Those gaining radon awareness earlier after moving into a given property were relatively younger, had more occupants per property, were more likely to be women, and have minors living at home (Fig. 1C,D).

For people who had just become aware of radon, we had previously noted^24^ differences between groups in ‘time to action’ (t_1_), best visualized by expressing number of months required to obtain a radon test as a function of the total number of radon awareness information interactions over the period (Fig. 1E). A majority of people (64.7%) required 2–5 interactions with radon awareness material over the course of a year before obtaining a radon test (denoted as ‘typical’); this contrasted with the 20.5% who displayed radon testing ‘urgency’ (obtaining a test in only a few weeks to months after a single encounter), or the 14.8% who exhibited ‘delaying’ behaviours (requiring > 6 interactions over many years). While there were no major demographic differences between the typical and urgent radon testing behavioural groups, the delaying group were substantially and significantly (p < 0.0001) over-represented by men, and were also somewhat less likely to have minors living in the property (Fig. 1F,G). For t_2_, the majority (92%) deployed their radon test within the first month of obtaining it, with no significant (p > 0.05) demographic differences to report compared to the minority who took longer (Fig. 1H). For t_3_ (radon testing period = 107.3 days) and t_4_ (time to receive outcomes = 19.2 days), there were also no clearly discernable differences between behaviours or demographic groups to report (Fig. 1I,J). From this analysis, we concluded that the largest differences driven by behaviour are based on time needed to first learn about radon (t_0_), and then the time taken to obtain a radon test (t_1_).

### Variable alpha particle radiation doses as a function radon awareness and testing behaviour

**Figure 2:**
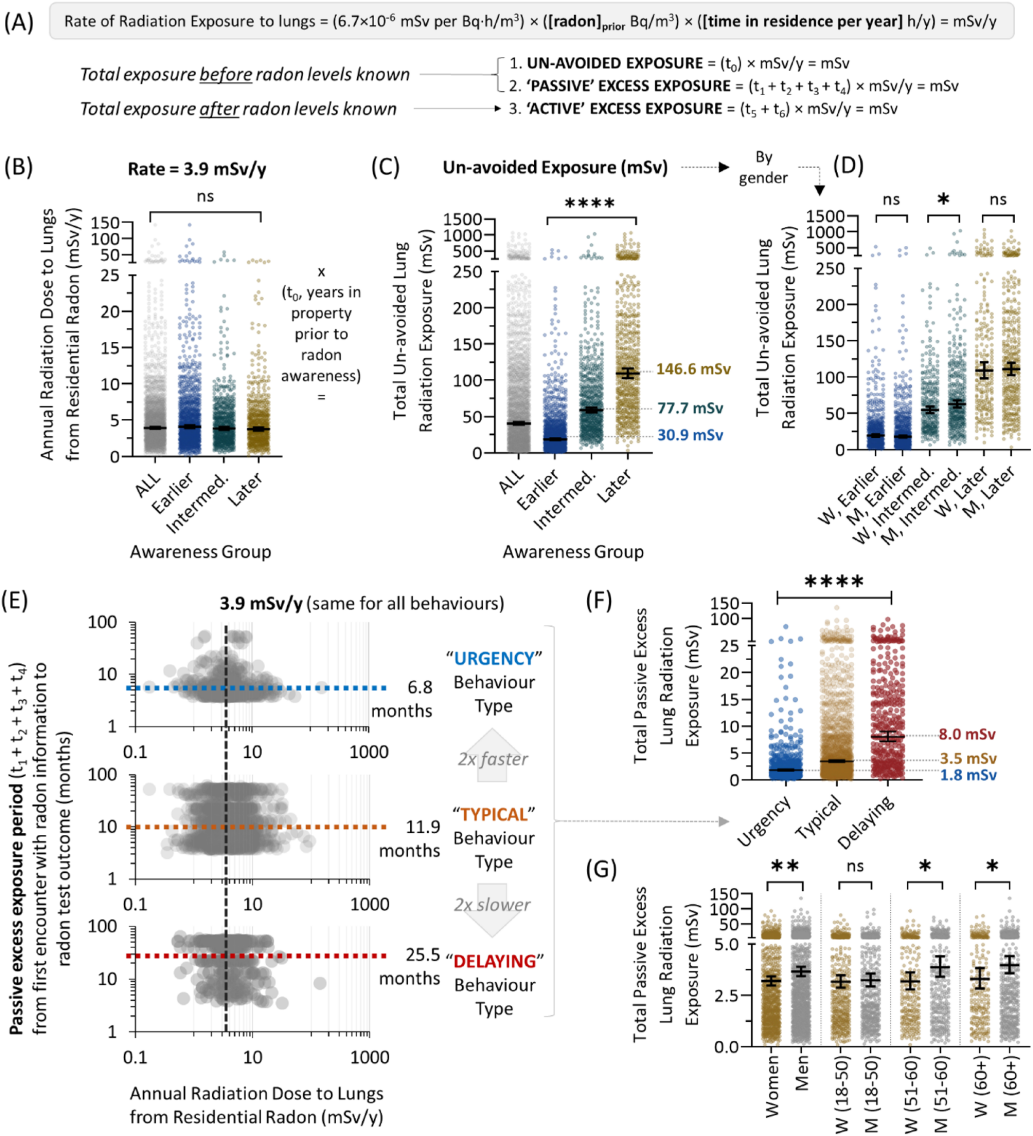
Variable doses of alpha particle radiation to the lungs as a function radon awareness and testing behaviour. (**A**) Conversion formulae and exposure group classifications. (**B**) Individual annual radiation doses to the lungs from residential radon for all participants, and as a function of initial awareness (t_0_) groups. The geometric mean rate of ‘un-avoided’ exposure for all groups was 3.9 mSv/year. (**C**) Total radiation exposures incurred during t_0_ for all participants and initial awareness groups. (**D**) Data from (**C**) separated by properties radon level, visualized by correlating the known radon level of their property with bracketed participant sex/gender. (**E**) Passive exposure periods (t_1+2+3+4_) for groups defined by radon test behaviour types. (**F**) Total radiation exposures incurred during (t_1+2+3+4_) as a function of radon test behaviour types. Data from (**F**) separated by participant age group and sex/gender. Statistical comparisons are Mann–Whitney pairwise nonparametric t-tests of comparisons for scatter plot data. ****p < 0.0001; ***p < 0.001; ns = p > 0.05. Figures were prepared using Excel and GraphPad Prism 9.1.1 (225) (www.graphpad.com).

Based on the distinct behavioural groups outlined above, we next calculated doses of alpha particle radiation to the lungs (in mSv) using our activity pattern data for all individuals and a conversion formula standardized by the ICRP (see “Methods” section, Fig. 2A). Three periods were considered: the un-avoided exposure period (all time prior to radon awareness, t_0_), the ‘passive’ excess exposure period (all time after general awareness of radon but before specific household radon levels are known, t_1+2+3+4_), and the ‘active’ excess exposure period (all time after personal radon exposure information is known, t_5+6_). For all participants, irrespective of behavioural group, the geometric mean rate of alpha radiation exposure due to residential radon was 3.9 mSv/year (min = 0.08; max = 157), demonstrating that household radon levels and activity patterns were random with respect to radon awareness and testing behaviour type (Fig. 2B). However, sizable differences emerged based on behaviour when total mSv radiation exposure was considered. For the total un-avoided excess exposure period (t_0+1_), 146.6 mSv was experienced by those who became aware of radon later after moving into the property, and this was significantly (p < 0.0001) and substantially (474%) greater than the 30.9–77.7 mSv total dose absorbed by those who gained awareness earlier (Fig. 2C). Within these groups, the only notable demographic difference to report was that un-avoided exposure doses were slightly but significantly (p < 0.05) higher in men versus women for the intermediate awareness group (Fig. 2D). For the total passive excess exposure period (t_1+2+3+4_), although the rate of radiation exposure from residential radon was identical (3.9 mSv/year) for all behavioural groups (i.e., random), differences in the total time from radon awareness to the receipt of household radon levels were clear. People displaying typical testing behaviour took, on average, 11.9 months from first hearing about radon to obtaining a radon test outcome for their property, resulting in 3.5 mSv excess exposure (Fig. 2E,F). By contrast, those exhibiting testing urgency obtained their household radon level in just 6.8 months (during which they experienced 1.8 mSv), while those who delayed required 25.5 months to receive their radon test outcome, during which they received 8.0 mSv alpha particle radiation to the lungs. Within these groups, men over age 50 experienced higher total passive excess exposure doses versus younger men, or women of any age (Fig. 2G).

### Reactions to a long-term residential radon test outcome

**Figure 3:**
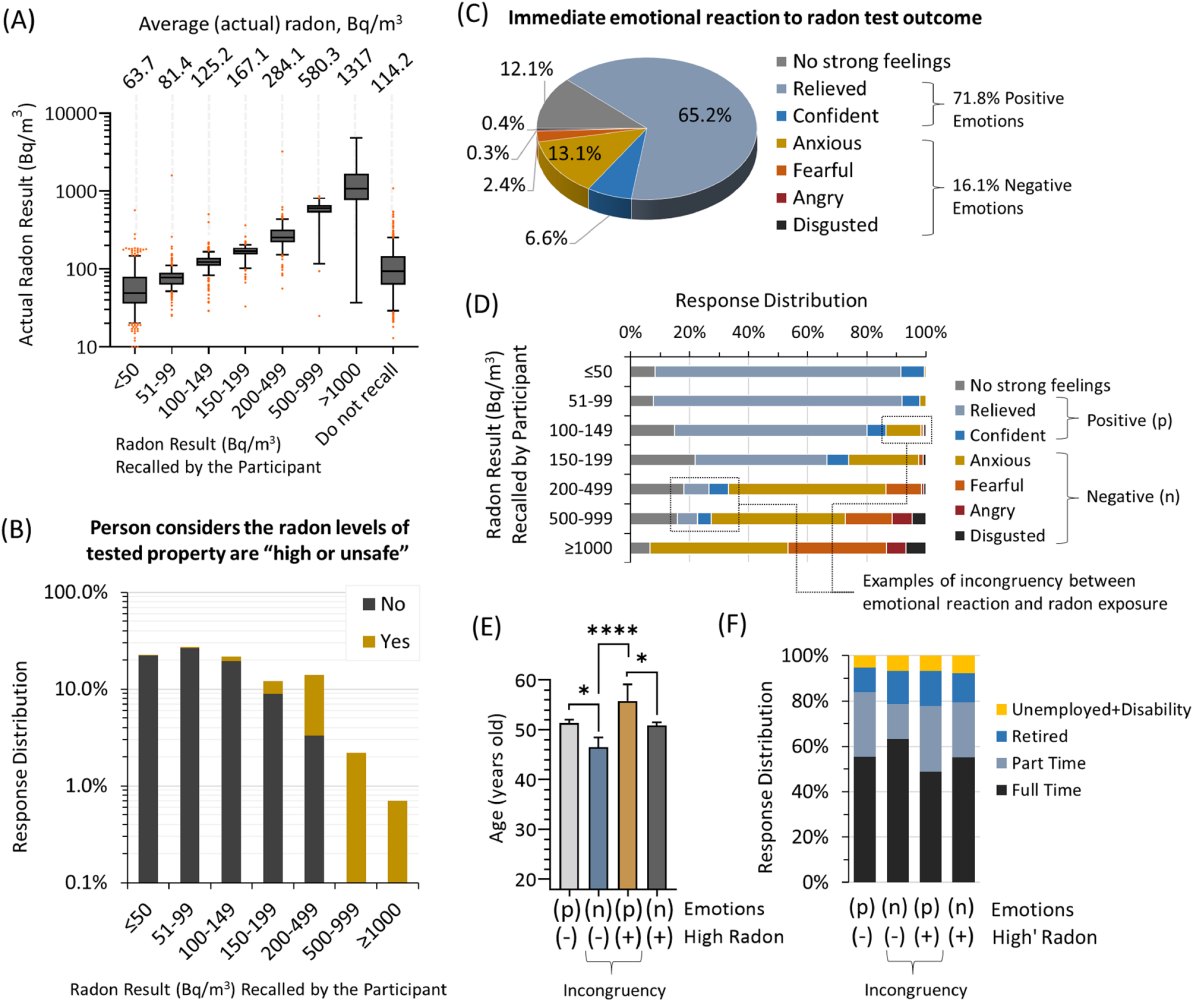
Recollection, interpretation, and emotional reactions to a long-term residential radon test outcome. (**A**) Box plot of the radon result (Bq/m^3^) recalled by the participant at the time of surveying, versus actual level of radon in the property. Data is expressed as quartiles, with whiskers = 5–95% and outliers denoted by orange dots. Values at the top of the graph denote the geometric mean of actual radon outcomes for each group. (**B**) Distribution of participant responses to whether they interpreted their radon test outcome as ‘high or unsafe’ or not, as a function of radon level recalled by participant. (**C**) Overall distribution of emotional responses to gaining direct knowledge of radon levels in a primary residence. (**D**) Responses from (**D**) as a function of radon result recalled by participant. Emotional reactions were considered incongruent if a person responded with either relief or confidence to a high radon outcome, or either anxiety, fear, anger or disgust at a low radon outcome. (**E**) Geometric mean ages of participants grouped as a function of their positive (p) or negative (n) emotional response as defined in (**D**), and whether or not they did (+) or did not (−) interpret their radon level as being high or unsafe (as defined in (**B**)). Statistical comparisons are Mann–Whitney pairwise nonparametric t-tests. ****p < 0.0001; *p < 0.05. (**F**) Using the groups described in (**E**), the distribution of full or part-time employment (or in education), retirement or unemployed/disability status of participants. Figures were prepared using Excel and GraphPad Prism 9.1.1 (225) (www.graphpad.com).

We next assessed how individuals were able to recollect and interpret the outcome of their radon test, and how they reacted emotionally to this information. To assess emotional reactions, we asked participants to reflect on their feelings and select an intensity based upon a standard 5-point psychometric Likert scale (as we had done previously^24^). The majority were able to correctly recollect their responses completed at the time of surveying (Fig. 3A). Perhaps expectedly, recall was the least precise for those whose household radon level was at or below 100 Bq/m^3^, as this level has been determined to be non-impacting to cancer risk^17,18^ (i.e., might be considered “unmemorable”). In terms of how this information was interpreted, all those with radon levels ≥ 500 Bq/m^3^ considered this level as high or unsafe, while the overwhelming majority (97%) with levels ≤ 150 Bq/m^3^ interpreted their level as low or safe (Fig. 3B). People with intermediate household radon outcomes nearer the regional, administrative radon action levels (of 200 Bq/m^3^) had mixed interpretations regarding safety, with 23.5% of those in homes with 200–499 Bq/m^3^ considering this as low or safe, and 25.8% of those in homes with 150–199 Bq/m^3^ considering this as high or unsafe. In total, 19.1% of participants interpreted their radon levels as high or unsafe, and this aligned with immediate emotional reactions (Fig. 3C), wherein 16.1% of participants expressed negative emotions (anxiety, fear, anger or disgust), 12.1% declared no strong feelings, and 71.8% experienced a positive emotion (relief or confidence) in a manner that was mostly congruent with their recalled radon result (Fig. 3D). A minority of participants emotional reactions were, at face value, incongruent with their recalled radon level, with 3.2% of people expressing positive emotional reactions to 200–999 Bq/m^3^ radon, and 3.1% experiencing anxiety in response to radon levels below 150 Bq/m^3^. To a small extent, this perceived incongruency correlated with age, with those reacting negatively to lower radon levels being younger or more likely to be in full time work or education, and those being more positive about higher radon being significantly (p < 0.0001) older or less likely to be in full time work or education (Fig. 3E,F).

### Intentions and radiation doses experienced by people interpreting their radon as low or safe

**Figure 4:**
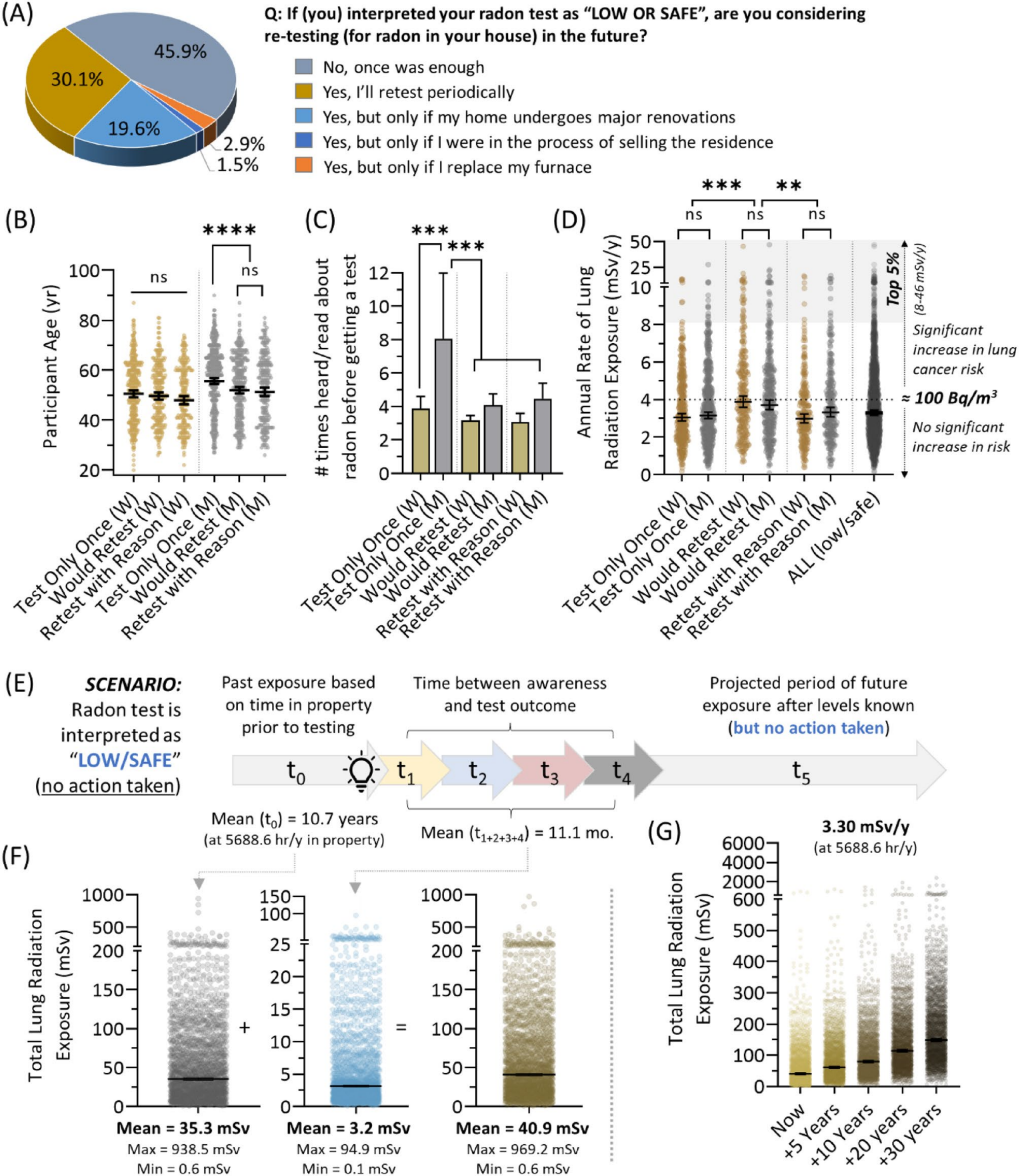
Behaviours and radiation doses to the lungs of people interpreting their radon as low or safe. (**A**) Distribution of stated intentions after gaining direct knowledge of primary residence radon levels, for those who interpreted their outcome as ‘low or safe’. (**B**) Distribution of participant ages as a function of sex/gender for each intention category based on (**A**). (**C**) For groups defined by (**A,B**), the number of times each needed to hear or read about radon before obtaining a radon test. (**D**) For groups defined by (**A,B**), the annual rate of lung radiation from residential radon experienced by individuals. (**E**) A schematic indicating the time periods (t_0_ to t_5_) influencing radon gas exposure in residential properties for which radon levels were interpreted as ‘low or safe’. t_0_ and t_1+2+3+4_ values = geometric means. (**F**) Scatter plots of total lung radiation experienced by individuals who identify their radon levels as ‘low or safe’. Mean values refer to geometric means. (**G**) Based on current (‘now’) total exposures (calculated in (**F**)), a projection of future total dose of radiation to lung from primary residential radon over a 5–30-year period, for those who interpret their radon levels as ‘low or safe’. Statistical comparisons are Mann–Whitney pairwise nonparametric t-tests of comparisons for scatter plot data. ****p < 0.0001; ***p < 0.001; **p < 0.01; ns = p > 0.05. Figures were prepared using Excel and GraphPad Prism 9.1.1 (225) (www.graphpad.com).

For the 80.9% of participants who interpreted their household radon level as low or safe, we queried whether they would consider radon testing at a future date. Almost half (45.9%) of people indicated no future intention to test (‘Test Only Once’), with the remainder split between 30.1% with periodic testing intentions (‘Would Retest’), and 24% who would only retest after either home renovations, furnace replacement, or in preparation for a real estate transaction (‘Retest with Reason’) (Fig. 4A). While there were no observable age effects between women in these groups, men with no future radon testing intention were significantly (p < 0.0001) older than men who would retest (Fig. 4B); this observation correlated with the same group initially requiring a greater number of interactions with radon awareness before obtaining their first radon test—indicative of consistently slower behaviour with regard to radon-related action (Fig. 4C). A possible explanation for why some expressed an intention to retest periodically without specific reason is that they had a modest but significantly (p < 0.0001) higher annual rate of radiation exposure from radon, indicative of comparatively greater household radon and/or time spent per year in the property relative to other groups (Fig. 4D).

To understand the long-term radiation exposure outcomes for those interpreting their household radon levels as low or safe, we next calculated total lung radiation exposures across all time periods in the behaviourally variable paradigm of radon awareness and testing outlined in Fig. 1A. Overall, the entire group of households with ‘low or safe’ radon had an average of 3.3 mSv/year (min = 0.08; max = 46.9) radiation exposure, with 59.7% being below the 4 mSv/year (≈ 100 Bq/m^3^) threshold for long term exposure at and above which increased lifetime risk of lung cancer risk is evident^17,18^ (Fig. 4D). As no radon reduction action was taken in the context of this ‘low/safe’ group (average household radon = 86.5 Bq/m^3^), time period t_6_ was excluded (Fig. 4E). The total un-avoided exposure was 35.3 mSv, based on an average of 10.7 years spent in the property prior to radon awareness, with a further 3.2 mSv during the 11.1-month passive exposure period, totalling to a geometric mean exposure of 40.9 mSv (Fig. 4F). Projecting forward at a mean rate of 3.3 mSv/year (and assuming no other variable changed), the population interpreting their household radon level as low or safe would accrue 61.5 mSv total radiation exposure from their household radon after 5 years, rising to a total of 148.9 mSv after 30 years (Fig. 4G). For context, these levels are only slightly below what would be accrued from the long term rate of irradiation from radon (4 mSv/year) that produces a 16% increase in relative lifetime risk of lung cancer^17,18^. It is important to note that 40.3% of households identifying their radon as ‘low or safe’ are, in fact, experiencing exposures rates in excess of 4 mSv/year, being as high as 8.0–46.9 mSv/year for most highly-exposed 5%, and receiving total doses of radiation that are predicted to increase lifetime relative risk of lung cancer over 10–30 years by a significant amount (Fig. 4D).

### Stated intentions of people interpreting their radon as high or unsafe

**Figure 5:**
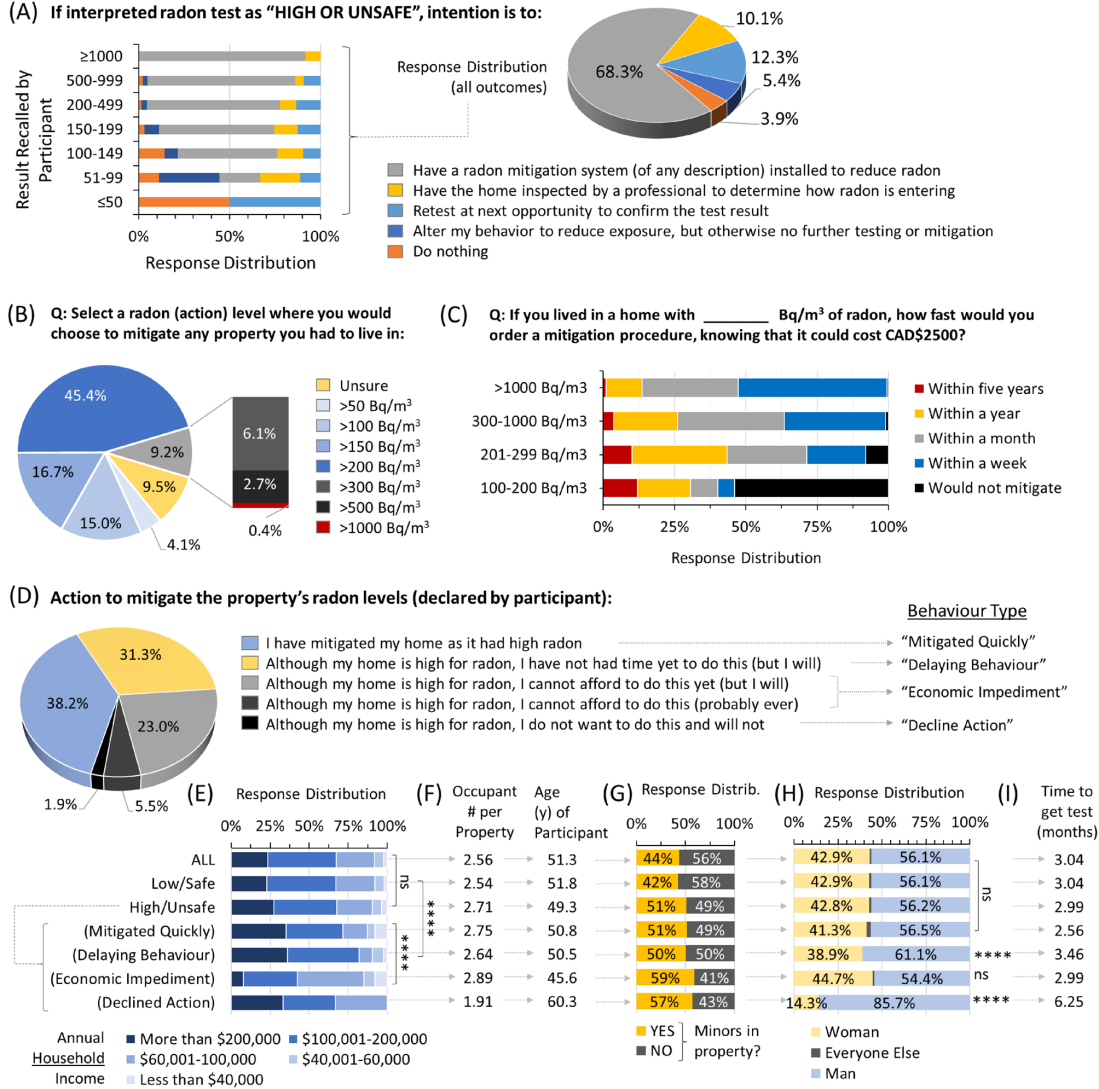
Behaviours and decision making of people interpreting their radon as high or unsafe. (**A**) Distribution of stated intentions after gaining direct knowledge of primary residence radon levels, for those who interpreted their outcome as ‘high or unsafe’, both overall (pie chart) and as a function of radon level (bar graph). (**B**) Distribution of responses to the radon level that a person would use as a threshold to guide a decision to obtain a professional radon mitigation. (**C**) Distribution of responses (as a function of radon level) to how fast an individual might order a radon reduction costing CAD$2,500 (a typical cost for a professional radon mitigation in Canada at the time of surveying). (**D**) Distribution of actions performed at the time of surveying, categorized into behaviour types. (**E**) Annual household income levels for all participants (ALL), those who either interpreted their radon level as ‘low/safe’ or ‘high/unsafe’, and also for those with ‘high/unsafe’ radon grouped by behaviour types outlined in (**D**). (**F**) Geometric mean occupants per property and age of primary respondent for groups defined in (**E**). (**G**) Sex/gender distribution for groups defined in (**E**). (**H**) Geometric mean time to obtain a radon test for groups defined in (**E**). (**I**) Response distribution to whether there were children living in property at time of radon testing, for groups define in (**E**). Statistical comparisons are 1-way ANOVA. ****p < 0.0001; ns = p > 0.05. Figures were prepared using Excel and GraphPad Prism 9.1.1 (225) (www.graphpad.com).

For the 19.1% of all participants who interpreted their household radon level as high or unsafe, we asked people to state their intended course of action (Fig. 5A). A majority (68.3%) expressed the intention to have a radon mitigation system (of any description) installed to reduce radon, with another 10.1% wishing to confer with a professional mitigator to determine how radon might be entering their home, and 12.3% desiring to confirm their radon levels via another test. A small (5.4%) group stated they might alter behaviour to reduce exposure, but otherwise take no other action, and only 3.9% of people declared they would take no action at all. When viewed in the context of radon level, most of those who stated that they would take no action (or only behaviour changes) largely fell into groups with lower radon (< 200 Bq/m^3^), with nearly everyone with higher radon levels indicating an intention to pursue more tangible radon reduction or retesting measures. This aligned well with stated action thresholds, wherein a majority (83.2%) chose to mitigate at levels in the 100–300 Bq/m^3^ range, and a small minority indicating their action threshold at very low (< 50 Bq/m^3^) levels (4.1% people) or very high (> 500 Bq/m^3^) levels (3.1% people) (Fig. 5B). To explore issues surrounding the socioeconomics of radon remediation, we next asked at what level of radon and how fast people would invest CAD$2500.00 in a property mitigation (Fig. 5C). This cost was chosen as it corresponded to empirically understood minimums for Canadian radon mitigation at the time of surveying. Somewhat unsurprisingly, both urgency and intentions to invest financial resources in mitigation corresponded with increased radon level, with a clear majority expressing a desire to mitigate radon levels within a week to a year of theoretically learning their property was over the regional action threshold of 200 Bq/m^3^.

### Behaviours and decision making of people interpreting their radon as high or unsafe

To understand actual behavioural choices and outcomes, we next captured the actual course of action taken by participants (Fig. 5D). More than a third (38.2%) declared they had completed (or had booked) a professional radon mitigation in response to a radon test outcome they interpreted as high or unsafe; we classified this group as those who ‘mitigated quickly’. Another 31.3% indicated the intention to mitigate, but that they had not yet had time; we classified this group as those who exhibited mitigation ‘delaying behaviour’. A total of 28.5% of people indicated that, although they desired mitigate their property for radon, they were not able to afford this either at the time of surveying or ever; we classified this group as those with ‘economic impediments’. Finally, 1.9% of people completely ‘declined action’. When interpreted through the lens of socioeconomics, it was clear that the group reporting economic impediments to radon mitigation had a substantially and significantly (p < 0.0001) lower household (before tax) income versus all other groups (Fig. 5E). This group were also younger, lived with a greater number of household occupants (Fig. 5F), were more likely to have minors living in the property (Fig. 5G), but were otherwise comparable to overall cohort in terms of the balance between men and women (Fig. 5H), or the time needed to initially obtain a radon test (Fig. 5I). Those who were able to obtain a mitigation more quickly encompassed somewhat older people with comparatively higher income levels than those who could not afford this, but were otherwise comparable in terms of the balance between men and women and other variables (Fig. 5E–I). Those who exhibited delaying behaviour encompassed those with the highest overall income levels, were significantly (p < 0.0001) over-represented by men, and overlapped with those individuals who required longer to obtain a radon test (Fig. 5E–I). Similarly, those who declined action altogether were overwhelmingly men, older, had fewer people living in the property and, originally, were those who took the longest time to obtain a radon test (Fig. 5E–I). For context, at the time of surveying, the average regional household income (before tax) was approximately CAD$118,000.00 according to *Statistics Canada*^31^. As our cohort encompassed 32.7% of households reporting before tax incomes under CAD$100,000.00 and 43.8% with between CAD$100,000.00 and CAD$200,000.00, the people participating in this study broadly reflected the income levels of the general population, albeit probabilistically somewhat skewed towards middle-income earners (Fig. 5E).

### Radiation doses experienced by people interpreting their radon as high or unsafe

**Figure 6:**
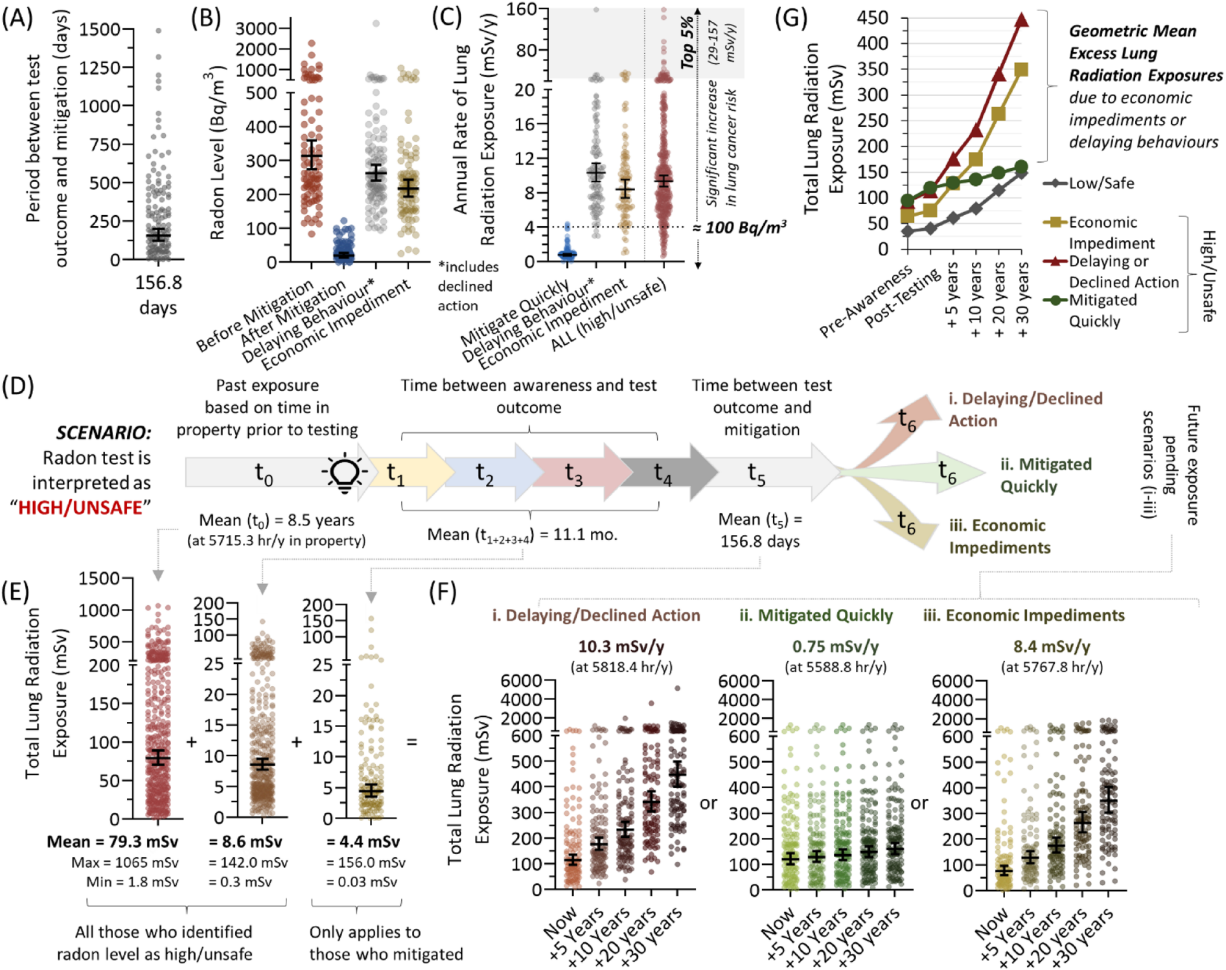
Radiation doses to the lungs of people interpreting their radon as high or unsafe, as a function of radon mitigation behaviour. (**A**) Individual (dots) and geometric mean (line and number) time periods between obtaining a radon test outcome to completion of a radon reduction mitigation, for those who interpreted their primary residential radon test outcome as ‘high/unsafe’ and who obtained a radon mitigation. (**B**) Residential radon levels for the behavioural groups outlined in Fig. 5D,E, including post-mitigation radon levels verified for those who ‘mitigated quickly’ (blue dots). (**C**) Annual rate of lung radiation exposure from radon for the behavioural groups from (**B**). Rates for the ‘mitigated quickly’ group were derived from post-radon mitigation levels. (**D**) A schematic indicating the time periods (t_0_ to t_6_) influencing radon gas exposure in residential properties for which radon levels were interpreted as ‘high or unsafe’ with three potential long-term outcomes depending on radon mitigation behaviour type. t values = geometric means. (**E**) Scatter plots of total lung radiation experienced by individuals who identify their radon levels as ‘high/unsafe’ for indicated exposure periods. Mean values refer to geometric means. (**F**) Based on current (‘now’) total exposures (calculated as the sum of exposures from (**E**)), and variable behavioural responses modifying future exposure (i.e., radon reduction or not), a projection of future total doses of radiation to lung from residential radon over a 5–30-year period. (**G**) Line graph of total lung radiation exposure data from (**E**) relative to long term exposure of those interpreting their household radon as ‘low/safe’. Figures were prepared using Excel and GraphPad Prism 9.1.1 (225) (www.graphpad.com).

To understand the long-term radiation exposure outcomes for those interpreting their household radon levels as high or unsafe, we next determined the amount of time between radon outcome and mitigation (t_5_), and the exact reduction in radon level that was achieved. For those that ‘mitigated quickly’, an average 156.8 days was needed (after receiving a test outcome) to complete a mitigation that reduced their properties geometric mean radon level from 313.5 to 19.2 Bq/m^3^ (Fig. 6A,B). The radon level for those who exhibited radon mitigation delaying behaviour or economic impediments remained high at geometric means of 262.4 and 216.7 Bq/m^3^, respectively. Overall, and prior to any mitigation, the entire group of households with ‘high or unsafe’ radon had an average of 9.3 mSv/year (min = 0.6; max = 157.8) radiation exposure, with 91.8% being above the 4 mSv/year (≈ 100 Bq/m^3^) threshold at which increased lifetime risk of lung cancer risk is evident^17,18^ (Fig. 6C). Those who were able to mitigate quickly reduced their annual exposure to 0.75 mSv/year. It is worth emphasizing that this level of exposure is well below the 4 mSv/year threshold of risk, and was substantially (440%) lower than the 3.3 mSv/year experienced by those identifying their household radon as ‘low or safe’ (Fig. 4D).

We next calculated total lung radiation exposures across all time periods in the behaviourally variable paradigm of radon awareness, testing and observed mitigation outcomes outlined in Fig. 6D. The total un-avoided exposure was 79.3 mSv, based on an average of 8.5 years spent in the property prior to radon awareness, with a further 8.6 mSv during the 11.1-month passive exposure period (Fig. 6E). For those who mitigated quickly, 4.4 mSv was experienced during the period between test outcome and mitigation, and their long-term exposure at 0.75 mSv/year resulted in a projected 5-year total radiation exposure of 128.5 mSv, rising to just 159.9 mSv after 30 years (Fig. 6F). For individuals that exhibited mitigation delaying behaviour or who declined action entirely, their long-term exposure was, on average, 10.3 mSv/year and resulted in a projected 5-year total radiation exposure of 176.6 mSv, rising to 447.3 mSv after 30 years. People unable to afford radon mitigation continued to be exposed to 8.4 mSv/year over the long term, which resulted in a projected 5-year total radiation exposure of 127.7 mSv, rising to 349.2 mSv after 30 years. When examined collectively, the behaviourally driven excess radiation exposure was largest for those with high radon and who delayed/declined action, being > 300 mSv greater compared to those who mitigated quickly or had lower radon (Fig. 6G). Those with economic impediments precluding mitigating their homes for radon experienced a > 250 mSv total excess exposure. Of note, while people who ultimately found that their homes contained ‘high/unsafe’ radon had already absorbed a far greater un-avoided radiation dose to their lungs at the time of first radon awareness (relative to those with low radon), this difference is predicted to be erased for those able to opt for mitigation, becoming indistinguishable from the low radon exposure group over time.

### Radiation exposure from radon as a function of integrated behaviours over time

**Figure 7:**
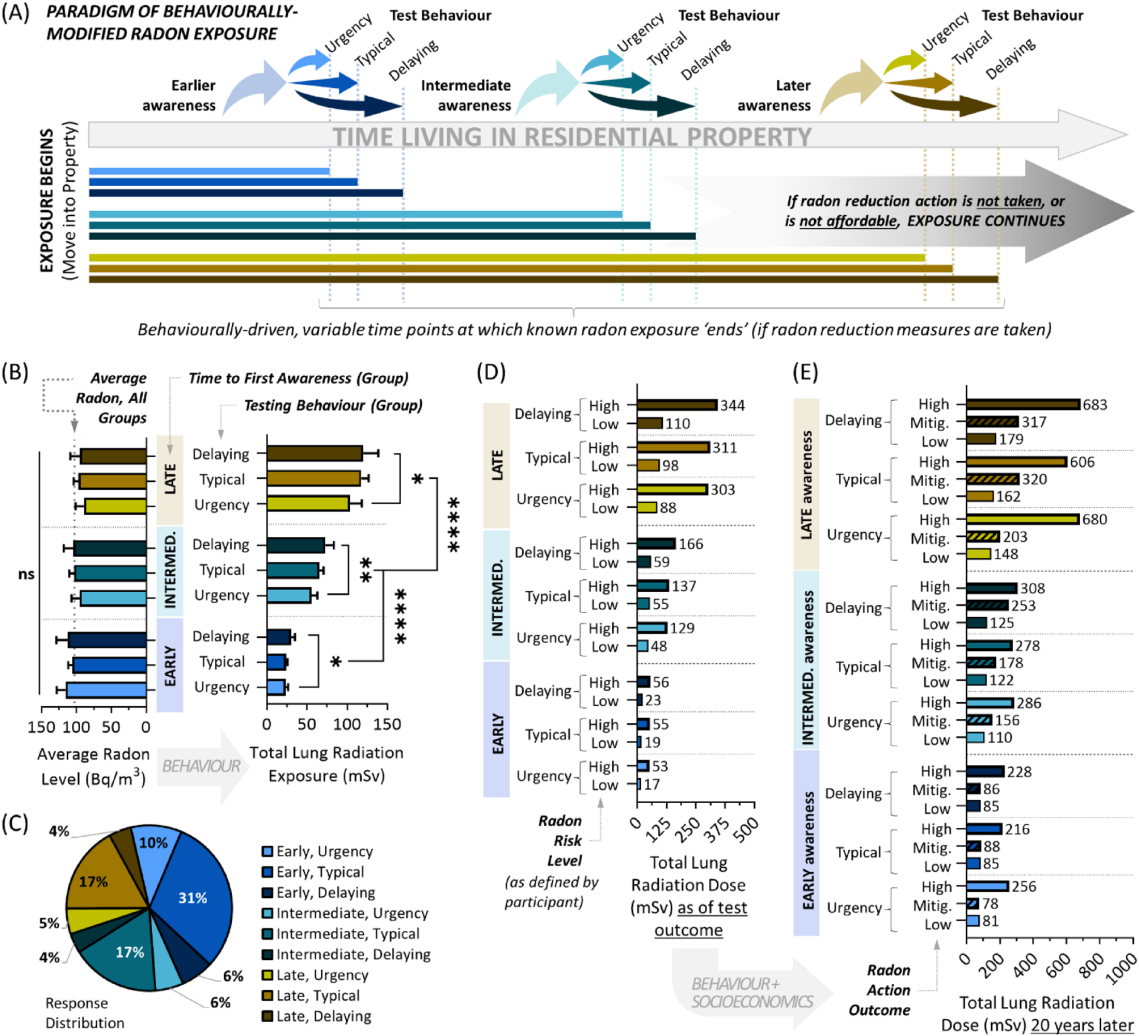
A paradigm for radiation exposure from radon as a function of integrated behaviours over time. (**A**) A schematic for all events and behaviours that modify doses of lung radiation from residential radon, independent of a property radon level. (**B**) The geometric mean property radon level (Bq/m^3^) and total lung radiation dose (mSv) experienced by nine indicated groups delineated by both period of radon awareness and radon testing behaviour. Statistical comparisons are Mann–Whitney pairwise nonparametric t-tests. ****p < 0.0001; **p < 0.01; *p < 0.05; ns = p > 0.05. (**C**) Relative distribution of the nine groups indicated in (**B**) within the participant cohort. (**D**) Actual geometric mean total lung radiation dose (mSv) experienced by the groups outlined in (**B**) at the time property radon level outcomes were known, as a function of risk level (interpreted by individual). (**E**) Projected geometric mean total lung radiation dose (mSv) experienced by the groups outlined in (**D**) 20 years later, separating those who interpreted their radon risk as high/unsafe into those who mitigated their property (‘mitig.’) or did not (‘high’). Figures were prepared using Excel and GraphPad Prism 9.1.1 (225) (www.graphpad.com).

Our findings so far indicate that there are three major behavioural types influencing the time periods that substantially modify absorbed radiation exposure due to residential radon. In this paradigm of behaviourally modified radon exposure (Fig. 7A), the major considerations are (i) how long it takes someone to first become aware of radon after moving into a property, (ii) the time needed to obtain a radon test and outcome, and (iii) whether or not (and how long it takes) an individual to reduce their radon exposure following a high or unsafe radon outcome. We next considered these variables holistically, and in relation to household radon levels, to determine if these integrated behaviours operated independently of the innate risks of radon exposure produced as a function of geology and the built environment. Strikingly, there was no significant difference in the radon level of the primary residence for the groups split apart by time to first radon awareness and radon testing behaviour, either relative to one another, or the geometric mean for the cohort as a whole (Fig. 7B). However, despite household radon levels being comparable, these groups experienced significantly (p < 0.01 to 0.0001) different total lung radiation exposures (Fig. 7B) based on their distributed behaviour types (Fig. 7C). By further factoring how individuals in these behavioural groups interpreted their household radon test outcome, further differences in total radiation exposure (at the time of radon test outcome) were evident, even for those with lower levels, and especially for those with high radon (Fig. 7D).

Finally, to estimate the added longer-term impact of radon reduction behaviours, we projected absorbed radiation doses forward over two decades for those who did or did not reduce high/unsafe radon, relative to the low radon group (Fig. 7E). Doing this, the importance of pursuing or being able to afford radon mitigation following a high radon test outcome was emphasized, as those who mitigated were able to ameliorate their exposures in a manner that offset previous behavioural effects. For example, an individual who became radon aware late (> 20 years after moving into their property), who then also delayed radon testing (taking > 2 years to get a test following awareness), and then who experienced a high radon test outcome but was able to mitigate their property for high radon quickly was, after a further 20 years, able to reduce their overall exposure to near that of a person who became radon aware earlier in life, who tested urgently, and then experienced high radon but did not (or were not able to) mitigate (317 versus 256 mSv, respectively). Collectively, these results confirm that social, economic, and behavioural differences between people are significant variables that modify radiation exposure from radon beyond the intrinsic exposure risks that are linked to and understood from household radon level information.

## Discussion

A key takeaway message from this work is that behavioural and social variables (particularly socioeconomic factors) have a strong influence on realized doses of radiation absorbed by the lungs as a consequence of radon in the residential built environment. These effects also operate in addition to how radon exposures are shaped by region (geology, climate, etc.), and the built environment (house age, type, etc.). This concept is important to consider in the context of population attributable risk of lung cancer from radon exposure, which has largely only been considered as a function of a property or regional radon level (and not group or individual level behaviours). As indicated earlier, 100 Bq/m^3^ radon exposure equates to ~ 4 mSv/year and carries the minimum, statistically significant increase (of 16%) in relative lifetime risk of lung cancer over the long term, and this value has been used in many regions as a reference point to derive administrative action levels (e.g., in Canada and the EU, the action level was set at twice where an elevated risk of cancer is first evident, i.e., 200 Bq/m^3^ radon). Risk estimations in this context are harmonized across populations irrespective of age, sex and gender, income, activity pattern, etc., and so are less suited for nuanced estimations of lung cancer risk at an individual level. To do this in a way that does not over or under-estimate risk, we suggest that mSv measures of radiation exposure be used, as these account for activity patterns and, in light of the information we present in this study, can also include and better reflect the clearly important impact of behaviours.

Personalized estimations of lung cancer risk from radon are increasingly important in a practical sense, as low-dose computed tomography is a clinically effective lung cancer screening tool being realized in many health systems, including Canada^2,32–34^. Two of the largest trials to date demonstrate a 20–24% reduction in lung cancer mortality using this approach^35,36^, with eligibility for screening derived from a ≥ 1.5% risk of lung cancer within a 6-year period. However, this eligibility criteria currently only applies to a proven history of heavy tobacco smoking (≥ 20–30 pack years). Indeed, light-, remote ex-, and never-smokers (and others with significant exposures to lung carcinogens) are currently excluded from lung cancer screening on the basis of insufficient evidence for benefit vs risk to support their inclusion based on health economics—this is an unaddressed health equity issue in all lung cancer screening programs. As it is anticipated that these programs will have a substantial positive impact on lung cancer outcomes, it is important to try and widen inclusivity while not jeopardizing their cost–benefit value. For radon to be used as a future lung cancer risk factor that confers screening eligibility, it will be very important to derive risk that holistically accounts for residential property radon level, overall human activity patterns, and the impact of behaviour on past, current and projected doses of alpha particle radiation to the lungs.

One striking observation we made was that, for those who interpreted their properties radon levels as ‘low or safe’, almost half declared no future intention to radon test again. In our study, long term (> 90 day) alpha track radon tests that were quality controlled by blanks, duplicates and spikes were used to ensure highly reliable readings with very little probability of false negative or positive outcome (verified and discussed in detail in Ref.^11^). However, in the more diverse context of global radon testing, it is important to consider that short term tests are available and even prevalent in some regions (e.g., the USA), and carry a proven^11^ much greater risk of false negative outcomes (i.e., the test appears low, while actual exposure is high). As half of those receiving a low reading will not test again, it follows that the long term consequences of any false negative radon test outcome on health will be amplified, potentially across a lifetime. Thus, we suggest that radon test reliability is an especially important consideration that all those offering radon tests to the public need to be concerned about. In this case, sensitivity, precision and accuracy should be prioritized over speed.

Some of the larger effects we documented in the behaviourally driven paradigm of radon exposure (Fig. 7A) occurred in a sex/gendered-, income-, and/or age-biased manner. It is important to recognize that our cohort encompassed people who served in the role of primary radon test decision maker for an entire household, in which an average of 2.56 people lived full time, including 44% of households with minors. Therefore, this is a scenario where the health-seeking behaviour of an individual can have wider implications on a group of people with potentially no or only indirect input into the decision. We consistently found that men (as the primary radon test decision maker) were more likely to become radon aware later in life and, once aware, require far more time and interactions with information before performing a test. Men were also more likely to delay or decline action following what they interpreted as a high or unsafe radon outcome, independent of their household income.

Hence, households where men are directing radon test decision making are, as a whole, likely to be exposed to alpha radiation from radon for longer, incurring greater risks over the long term. This effect was, in part, skewed by age, with men who were under age 50 (at the time of surveying) displaying improved health-seeking behavior (or, at least, distinct structural determinants of health). This has important implications for the targeting of radon testing and mitigation measures, and demonstrates that attempting to engage with younger people or women of any age in risk communication strategies could show a benefit to both deciding to mitigate and doing so more quickly. This phenomenon has precedent in health-seeking behaviour differences by sex and gender more broadly, in that it has been demonstrated across the behavioural and healthcare experiences of men and women^37^. One acknowledged caveat to our work is that we were not able to obtain gender identity information from all participants and, in those cases, deferred to reported sex. It will be important in future work to obtain bona fide gender identity data from a wider population. Similarly, only 1% of our cohort identified with a gender minority, and so we were unable to analyse this group for most outcomes reported here. Future work will be needed to determine effects for the diversity of gender minorities, as well as a function of other demographic factors such as ethnicity and acculturation.

In addition to behaviour, our work demonstrates that a substantial excess exposure to alpha radiation from radon corresponds with the inability to afford radon mitigation, despite the desire to do so. As this situation was more likely to be experienced by younger people with the most people (and minors) living in the household, this is very undesirable from a cancer prevention and health justice perspective. One possible solution to this would be to subsidize radon mitigation costs for homeowners under a certain income level, or those with younger children in the property. Landlords (whether individuals or corporations) might also be encouraged or mandated to ensure rental properties do not contain high radon levels, conferring protection to a wider population of people who are not homeowners. Although undoubtedly complex to enact or enforce, costs would be offset by an increase in successful radon mitigation outcomes following a test interpreted as high or unsafe. In this cohort, mitigation rates were 38.2%, but could rise to 66.7% if those who expressed the desire to mitigate but could not afford to were enabled to do so. We acknowledge that enabling people to mitigate for radon may not purely be an economic issue, and that there are likely other structural determinants of health at play that have yet to be clearly identified. For example, our study was unable to address inequities for Indigenous North American populations who may experience income instability coupled with remoteness (potentially reduced internet access), and lack of tailored messaging and resources to address radon mitigation. This is a particular gap that must be addressed in nations with populations experiencing marginalization. The best solution to this entire problem would be to enact new building codes so that new properties would be required to be ‘radon-safe’, and require mitigation strategies in all existing properties at a policy/regulatory level, which would be government/industry subsidized for people unable to afford or carry this out.

Finally, we found that those in properties high for radon, and who also were able to mitigate those houses, had, overall, the lowest possible rates of radon exposure of all groups moving forward. Strikingly, those in high radon-mitigated households were able to achieve 440% reductions in annual average alpha particle radiation exposures, to levels considered low even compared to those in houses where radon tests were interpreted as ‘low or safe’, at only 0.75 mSv/year, a level that is not believed to have any measurable impact on lung cancer risk^14,17,18,22^. Conversely, an alarming percentage of those who interpreted their radon test as ‘low or safe’ in fact experienced exposure rates far in excess of the 4 mSv/year threshold where increased relative risk of lung cancer has been established, reaching > 40 mSv/year in extreme cases. Our work suggests that this, in part, is driven by regional radon action thresholds (i.e., 200 Bq/m^3^) that are used by individuals to interpret relative safety, and is further evidence that use of administrative thresholds that exceed levels where a statistically significant increase in lung cancer risk is evident (i.e., 100 Bq/m^3^) may be driving radon exposures the are, in fact, causing lung cancers across populations^13^. We speculate that lowering administrative action thresholds to 100 Bq/m^3^ would improve overall health-seeking behaviours and decision-making, encourage greater uptake on radon mitigation, and reduce the future burden of radon-induced lung cancers.

## Methods

### Statement of approvals

All activities were pre-approved by the Conjoint Health Research Ethics Board, Research Services, University of Calgary (IDs = REB17-2239, REB19-1522) or the Health Research Ethics Board of Alberta, Cancer Committee (HREBA.CC-17-0246), adhering to citizen science research best practice^38^, and in accordance with all regional guidelines and regulations.

### Participant eligibility and enrollment

Enrollment was based on convenience recruitment for all wanting to join, with all adult homeowners and renters in any residential building type being eligible. No data from any constituent part of this cohort were from known or pre-selected lung cancer cases. Commercial offices or hospitality service buildings were not considered. Records of informed consent were obtained in all cases. Participants were permitted to withdraw at any time.

### Radon awareness communication

Communication methods for study recruitment included dissemination of radon awareness information and provision of opportunities for radon testing through print media, public seminars, online (website and social media) and traditional mass media via organic (unpaid) TV, magazine, and radio exposure in an untargeted manner. Each of these points of communication was further boosted in an organic (unpaid) manner by health agencies, non-profit groups, and some private organizations. Radon awareness content included basic, scientifically informed facts on radon, its health effects in the context of lung cancer and highlighted both historic facts, figures, and emerging regional statistics and have been outlined in detail in Ref.^24^.

### Radon testing

Radon tests were quality controlled by Canadian National Radon Proficiency Program (C-NRPP) certified professionals and distributed centrally by researchers. Care was taken to educate participants in the correct test deployment methods through print, video and direct communication with C-NRPP-certified professionals who adhere to Health Canada’s guidelines for long-term (90+ day) radon testing. Participants were advised to place devices on the lowest level of the building occupied for approximately 4 or more hours per day. As participants positioned kits in areas and/or floors of the property where occupants spent the majority of their time, we did not apply any correction factor(s). Participants were asked to deploy radon tests within residences for a minimum of 3 months. Readings based on less than 60 full days were excluded from analysis and, for this cohort, the average period of radon testing was 107.8 days. Radon tests were RadTrak2 closed passive etched track detectors made from CR-39 plastic film inside antistatic holders enclosed in electrically conductive housing with filtered openings to permit gas diffusion, intended for long term (> 90 day) use with a typical linear range of 15 to 25,000 Bq/m^3^. To be read, CR-39 films are etched in 5.5 N NaOH at 70 °C for 15.5 min and scored using TrackEtch software at ISO17025 certified Radonova laboratories (Sweden, EU). Precision and accuracy controls have been reported previously in Ref.^11^.

### Surveying

From 2015 to 2020, Canadians purchased alpha track 90+ day radon detectors that they then deployed, returned for analysis, and later received their specific radon reading in a confidential manner. Radon outcomes for this cohort were reported most recently in Ref.^20^. Non-profit study kits were $51.99. Following consent and placement of a radon test, all participants active within the study in October of 2018 were invited to complete demographic surveys and questionnaires on radon awareness and testing experiences using the Qualtrics survey platforms. In November of 2020, all active participants were invited to complete further demographic surveys that included gender identity questions. Of all participants, we obtained both sex and gender identity data from 68.5, with 25% providing only sex data, and 6.5% declining to indicate either. As only 1% of participants chose a gender identity option that was not ‘woman’ or ‘man’, we were unable to analyse distinct gender or sex minority groups in greater detail, but rather identified people in this group as ‘everyone else’ for purely practical purposes in this study. A complete list of survey questions is outlined in Supplemental Information, Sect. I.

### Radiation exposure calculations

To convert Bq/m^3^ indoor air radon levels to human mSv radiation exposures (to lungs), the ICRP provides the following formula^39^:

$$\left( {6.7 \times 10^{ - 6} \,{\text{mSv }}per{\text{ Bq}} \cdot {\text{h}}/{\text{m}}^{3} } \right) \, \times \, \left( {\left[ {radon} \right]{\text{ Bq}}/{\text{m}}^{3} } \right) \, \times \, \left( {\left[ {time \, in \, residence \, per \, year} \right]{\text{ h}}/{\text{year}}} \right) \, = {\text{ mSv}}/{\text{year}}{.}$$

Values for the amount of time spent in the primary residence per year for a typical adult (*“time in residence per year”)* were calculated from individually reported residential occupancy data from 1128 participants, and cross-referenced with data from the *National Human Activity Pattern Study* (NHAPS)^21^. The NHAPS, which included responses from both Canadian and American respondents, estimated that 68.7% of life was spent inside a residence for the average adult. Of the total 8760 h per year, this equates to 6018 h/year and represents an average of all different employment statuses. Participants reported their data by season (Winter, Spring, Summer, Fall), with weekend/holiday versus workdays accounted for within the questionnaires described in detail in Ref.^20^. All response-derived “*time in residence per year”* outcomes were linked to individually reported employment statuses and used to extrapolate the same values for all remaining participants (2390) for which employment data was collected. Please note that for the purposes of participant responses in this survey, being enrolled in full or part time formal education (at a university, college, technical school, etc.) was considered to be a type of employment and grouped with those responses.

### Statistical analysis

Statistical analyses were carried out using Excel and GraphPad Prism 9.1.1 (225) (www.graphpad.com). One-way ANOVAs were carried out to test differences between groups (e.g., year of construction, occupant age, mSv, etc.), with Bonferroni–Holm corrected post-hoc testing carried out to characterize group differences for pairwise comparisons if the ANOVA reached significance. Mann–Whitney pairwise nonparametric t-tests were used to assess the significance of scatter plot data.

## Supplementary Information

Supplementary Information.

## Data Availability

The de-identified raw data sets generated by the current study are available to academic researchers following reasonable requests to Dr. Goodarzi and will require a legally binding data transfer agreement. Data may not be used for private, commercial, or for-profit purposes for any reason.
