# Supplementary material for: Social factors and behavioural reactions to radon test outcomes underlie differences in radiation exposure dose, independent of household radon level
Source: Sci Rep. 2022 Sep 14;12:15471. doi: 10.1038/s41598-022-19499-5 (PMC9473468; doi:10.1038/s41598-022-19499-5)
Supplement: Supplementary file 1 — Supplementary Information. [file 41598_2022_19499_MOESM1_ESM.pdf]

SUPPLEMENTAL FILE

for

Social factors and behavioural reactions to radon test outcomes underlie differences in radiation exposure dose, independent of household radon level

Jesse L. Irvine[1*], Justin A. Simms[2*], Natasha L. Cholowsky[1], Dustin D. Pearson[1], Cheryl. E. Peters[3], Linda E. Carlson[4**], Aaron A. Goodarzi[1**]

Author Affiliations:
[1]Robson DNA Science Centre, Departments of Biochemistry & Molecular Biology and Oncology, Charbonneau Cancer Institute, Cumming School of Medicine, University of Calgary, Alberta, Canada; [2]Faculty of Medicine, University of Saskatchewan, Saskatoon, Saskatchewan, Canada; [3] British Columbia Centre for Disease Control, British Columbia Cancer Agency, School of Population and Public Health, University of British Columbia, British Columbia, Canada. [4]Division of Psychosocial Oncology, Department of Oncology, Charbonneau Cancer Institute, Cumming School of Medicine, University of Calgary, Alberta, Canada.

*These authors contributed equally.

**Co-Corresponding authors are: L. Carlson (l.carlson@ucalgary.ca) and A. Goodarzi (a.goodarzi@ucalgary.ca)

Survey Questionnaire

The questions below were asked of participants following confirmation of informed consent using an online platform (Qualtrics) compatible with desktop and mobile devices.

Section 1 – Decision-making to test for radon gas

1. Approximately how many times did you hear or read about radon, before deciding to get a radon test for your property(s)?

2. Approximately how long was it from first hearing about radon to registering for or obtaining a radon test kit?

3. How long did it take you from the time you received your radon test, to setting it up in your home?
    a. Less than a week
    b. Less than a month
    c. Within 3 months
    d. Within 6 months
    e. Within a year
    f. Did not deploy yet
    g. Do not plan to deploy it/changed my mind

Section 2 – Reactions to Radon Test Results

1. How long have you lived in the home that you tested for radon?

2. What range did your radon test result fall into?
    a. 50 Bq/m^3 or less
    b. 51-99 Bq/m^3
    c. 100-149 Bq/m^3
    d. 150-199 Bq/m^3
    e. 200-499 Bq/m^3
    f. 500-999 Bq/m^3
    g. 1000 Bq/m^3 or more
    h. I don’t know/can’t remember
3. What was your immediate emotional reaction when you first received your radon test results?
    a. No strong feeling
    b. Relieved
        I. Extremely
        II. Very

        III. Moderately
        IV. Slightly
        V. Not at all
    c. confidant
        i. Extremely
        ii. Very
        iii. Moderately
        iv. Slightly
        v. Not at all
    d. Anxious
        i. Extremely
        ii. Very
        iii. Moderately
        iv. Slightly
        v. Not at all
    e. Fearful
        i. Extremely
        ii. Very
        iii. Moderately
        iv. Slightly
        v. Not at all
    f. Angry
        i. Extremely
        ii. Very
        iii. Moderately
        iv. Slightly
        v. Not at all
    g. Disgusted
        i. Extremely
        ii. Very
        iii. Moderately
        iv. Slightly
        v. Not at all

4. Did you know how to interpret your radon test results, or were able to get this information to your satisfaction?
    a. Yes
    b. Somewhat
    c. No

5. Do you believe that your radon levels are considered “high or unsafe”?
    a. Yes
    b. No

6. IF you interpret your radon test as “high or unsafe”, are you considering any of the following:
    a. Retesting at next opportunity to confirm the test result (YES/NO)
    b. Having the home inspected by a professional to determine how radon is entering
        i. Will do / did this within a few weeks or less
        ii. Will do / did this within a few months
        iii. Will do / did this next year / at some point in future
    c. Having a radon mitigation system (of any description) installed to reduce radon
        i. Will do / did this within a few weeks or less
        ii. Will do / did this within a few months
        iii. Will do / did this next year / at some point in future
    d. Altering my behavior to reduce exposure, but otherwise no further testing or mitigation
    e. Doing nothing:
        i. I do not know what to do about this, at present
        ii. I do not want to do anything about this, at present
        iii. I am not in a financial position to do anything about this, at present
        iv. I do not have time to do anything about this, at present
    f. Other
7. IF you interpret your radon test as “low or safe”, are you considering re-testing in the future?
    a. Yes, I’ll re-test periodically
    b. Yes, but only if my home undergoes major renovations
    c. Yes, but only if I replace my furnace
    d. Yes, but only if I were in the process of selling the residence
    e. No, once was enough

Section 3 – Decision to Mitigate for High Radon

1. Have you ever taken action to mitigate high radon in any property?
    a. No
    b. Yes (if so, why?)
        i. Concern for your health, or that of the others in the home
        ii. Concern for your ability to sell your home in the future
        iii. Other

2. Select a radon level where you would choose to mitigate any property you had to live in:
    a. More than 50 Bq/m^3
    b. More than 100 Bq/m^3
    c. More than 150 Bq/m^3
    d. More than 200 Bq/m^3
    e. More than 300 Bq/m^3
    f. More than 500 Bq/m^3
    g. More than 1000 Bq/m^3

h. I have no idea as I don't really understand what these mean

3. If you lived in a home with >1,000 Bq/m^3 of radon (considered very high radon levels; Health Canada's recommended action level is 200 Bq/m^3 [10]), how fast would you order a mitigation procedure, knowing that it could cost $2500?
    a. Within a week
    b. Within a month
    c. Within a year
    d. Within five years
    e. I would not mitigate

4. If you lived in a home with 300-1000 Bq/m^3 of radon (Health Canada's recommended action level is 200 Bq/m^3 [10]), how fast would you order a mitigation procedure, knowing that it could cost $2500?
    a. Immediately
    b. Within a week
    c. Within a month
    d. Within a year
    e. I would not mitigate

5. If you lived in a home with 201- 299 Bq/m^3 of radon (Health Canada's recommended action level is 200 Bq/m^3 [10]), how fast would you order a mitigation procedure, knowing that it could cost $2500?
    a. Immediately
    b. Within a week
    c. Within a month
    d. Within a year
    e. I would not mitigate

6. If you lived in a home with 100-200 Bq/m^3 of radon (Health Canada's recommended action level is 200 Bq/m^3 [10]), how fast would you order a mitigation procedure, knowing that it could cost $2500?
    a. Immediately
    b. Within a week
    c. Within a month
    d. Within a year
    e. I would not mitigate
7. Based on your radon results (obtained through our study), have you taken any action to mitigate the property's radon levels?
    a. Yes, I have mitigated my home as it had high radon
    b. No, I do not need to as my home read low for radon
    c. No, although my home is high for radon, I have not had time yet to do this (but I will)
    d. No, although my home is high for radon, I cannot afford to do this yet (but I will)

e. No, although my home is high for radon, I cannot afford to do this (probably ever)
f. No, although my home is high for radon, I do not want to this and will not
    i. I feel that radon mitigation will harm my home
    ii. I feel that radon mitigation is just too expensive
    iii. I feel that radon risks are exaggerated

8. When* was the radon mitigation work completed on the residence tested with the Evict Radon study? *If you do not remember a specific day exactly, please put the first of the month you select.
DD/MM/YYYY

9. Was the residence tested with the Evict Radon study ever later re-tested for radon?
    a. Yes
    b. No

10. What was the date* when the residential property was last re-tested for radon?
*If you do not remember a specific day exactly, please put the first of the month you select.
DD/MM/YYYY

11. What was the new radon level in Bq/m^3?

Section 4 – Activity Pattern (needed to calculate absorbed radiation)

What was your status in terms of employment or enrollment in education**?

a. Full-time or part-time employed and/or enrolled in education**
**These activities may take place at an office, school, or from your property.
b. Unemployed and/or not enrolled in education
c. Retired
d. On long-term leave (including parental leaves, medical leaves, disability, etc.)
e. Prefer not to say

How many *hours per day* in each season did you typically spend:

	**INSIDE** your residence (tested for Radon)	**INSIDE** a different residence	**INSIDE** a non-residential building (an office, store, business, school etc.)	**OUTDOORS**	Total
Winter (Dec, Jan, Feb)	hours per day	hours per day	hours per day	hours per day	hours per day
Spring (Mar, Apr, May)	hours per day	hours per day	hours per day	hours per day	hours per day
Summer (Jun, Jul, Aug)	hours per day	hours per day	hours per day	hours per day	hours per day
Fall (Sep, Oct, Nov)	hours per day	hours per day	hours per day	hours per day	hours per day

Section 5 – Demographics

1. What is your age (as of this year)?

2. What sex were you assigned at birth?
   a. Male
   b. Female
   c. Prefer not to say

3. What is your current gender identity? Please select all that apply.
   a. Man
   b. Woman
   c. Transgender
   d. Genderqueer/gender non-conforming
   e. Nonbinary
   f. Prefer to self-describe: ______
   g. Prefer not to say

4. What was your approximate, average HOUSEHOLD INCOME* over the past year**?

* *Household income is defined as the combined gross income of all adult members of the same primary residence. Individuals do not have to be related in any way to be considered members of the same household, although, if you have roommates or tenants who you do not know very well / are 'not part of your life', it is ok not to consider them in your response*

** *We are gathering this information as household income is a relevant determinant for socioeconomic status, which impacts health system access, risk awareness, as well occupancy trends within the residential environment.*

   a. No income or loss
   b. Less than $10,000
   c. $10,001 to $20,000
   d. $20,001 to $30,000
   e. $30,001 to $40,000
   f. $40,001 to $50,000
   g. $50,001 to $60,000
   h. $60,001 to $80,000
   i. $80,001 to $100,000
   j. $100,001 to $200,000
   k. More than $200,000
   l. Unsure or prefer not to say

5. As of this date, and including yourself in the count, how many adults (18 or more years old) live regularly in your residence?

6. As of this date, how many minors (0 – 17 years old) live regularly in your residence?

7. What was your relationship status at the time you decided to obtain a radon test?

a. Single, never married
b. Single, divorced or separated
c. Long term relationship, unmarried
d. Long term relationship, married or civil partnership (cohabiting)
e. Widow/er

8. How many <u>children</u> did you have at the time you decided to obtain a radon test?

f. 0
g. “0” (but I was/we were pregnant with a first child, or expecting to adopt)
h. 1
i. 2
j. 3
k. 4 or more
